# Efficacy of Crataegus Extract Mixture on Body Fat and Lipid Profiles in Overweight Adults: A 12-Week, Randomized, Double-Blind, Placebo-Controlled Trial

**DOI:** 10.3390/nu16040494

**Published:** 2024-02-08

**Authors:** Jungbin Song, Do-Yeon Kim, Han Songyi Lee, Sang Youl Rhee, Hyunjung Lim

**Affiliations:** 1 Department of Herbal Pharmacology, College of Korean Medicine, Kyung Hee University, 26 Kyungheedae-ro, Dongdaemun-gu, Seoul 02447, Republic of Korea; jbsong@khu.ac.kr; 2 Research Institute of Medical Nutrition, Kyung Hee University, 26 Kyungheedae-ro, Dongdaemun-gu, Seoul 02447, Republic of Korea; rou_@naver.com (D.-Y.K.); flowerlhsy@hanmail.net (H.S.L.); 3 Center for Digital Health, Medical Science Research Institute, Kyung Hee University Medical Center, 26 Kyungheedae-ro, Dongdaemun-gu, Seoul 02447, Republic of Korea; 4 Department of Endocrinology and Metabolism, College of Medicine, Kyung Hee University, 26 Kyungheedae-ro, Dongdaemun-gu, Seoul 02447, Republic of Korea; 5 Department of Medical Nutrition, Graduate School of East-West Medical Science, Kyung Hee University, 1732 Deogyeong-daero, Giheung-gu, Yongin-si 17104, Gyeonggi-do, Republic of Korea

**Keywords:** *Crataegus pinnatifida*, *Citrus unshiu*, Crataegus extract mixture, obesity, overweight, body fat, lipid, leptin

## Abstract

A Crataegus Extract Mixture (CEM) is a combination of extracts from *Crataegus pinnatifida* leaves and *Citrus unshiu* peels, well-known herbs used for treating obesity and dyslipidemia. We aimed to investigate the efficacy and safety of a CEM on the body fat and lipid profiles in overweight adults. A 12-week, randomized, double-blind, placebo-controlled, parallel-group trial was conducted on 105 subjects aged 20–60 years with body mass indexes between 25 and 30 kg/m^2^. Eligible subjects were randomly assigned in a 1:1:1 ratio to receive either a high dose of the CEM (400 mg tid), a low dose of the CEM (280 mg tid), or a placebo. Body fat was evaluated using dual-energy X-ray absorptiometry (DXA), bioelectrical impedance analysis (BIA), and anthropometric measurements. The blood lipid and adipokine profiles were measured before and after the administration. After 12 weeks, the reductions in the fat percentages measured by DXA and BIA were significantly greater in the CEM groups than in the placebo group. The CEM also significantly decreased the body weights, body mass indexes, and blood leptin levels. An additional per-protocol analysis revealed that the high dose of the CEM also lowered the blood levels of triglycerides and very low-density lipoprotein cholesterol. No adverse events occurred after the CEM treatment. Our results suggest that CEMs are safe and effective for reducing the body fat and body weight and regulating the blood lipid and leptin levels in overweight or mildly obese individuals.

## 1. Introduction

Obesity has become a public health threat that has reached epidemic proportions worldwide. Even in South Korea, where the prevalence is lower than in the Americas or Europe [1], overweight and obese adults with body mass indexes (BMIs) of 25 kg/m^2^ or more reached about 40% of the total population in 2020 [2]. Because obesity increases the risk of various cardiovascular, metabolic, and skeletal diseases and cancer [3,4], early intervention for weight management is needed from the overweight stage.

Approaches for weight loss are multidisciplinary, including lifestyle changes, functional foods/nutraceuticals, pharmacotherapy, bariatric surgery, etc. The currently approved anti-obesity medications (semaglutide, liraglutide, naltrexone/bupropion, phentermine/topiramate, orlistat) are indicated for patients with BMIs of ≥30 kg/m^2^ or of ≥27 kg/m^2^ with one or more comorbidities [5]. Overweight persons with BMIs below the thresholds are commonly prescribed costly long-term medications in an off-label manner [6]. Historically, many anti-obesity drugs have been withdrawn due to cardiotoxicity, psychiatric disturbances, or drug abuse and dependence [7], and, most recently, lorcaserin was withdrawn in 2020 due to the increased risk of cancer. In terms of safety and economy, alternative treatments, especially of plant origin, have attracted significant research interest. For overweight or mildly obese individuals, the use of traditional medicinal herbs with known efficacy and safety for weight loss can be considered rather than off-label drug use.

A Crataegus Extract Mixture (CEM) is an extract mixture of *Crataegus pinnatifida* leaves and *Citrus unshiu* peels. In East Asia, *C. pinnatifida* and *C. unshiu* have traditionally been used to promote digestion and relieve gastric food retention, and they are now widely used to treat obesity and dyslipidemia [8,9,10]. The beneficial effects of *C. pinnatifida* leaves and *C. unshiu* peels on fat reduction and lipid metabolism have been well documented in obese animals [8,9,11]. In addition, *C. unshiu* peels decreased the BMIs and lowered the blood levels of total cholesterol (TC) and triglycerides (TGs) in overweight and obese adults [12]. CEMs have been shown to reduce the visceral fat and regulate the blood lipid levels in high-fat-diet-fed rodents by preventing intestinal lipid absorption; decreasing hepatic lipogenesis and gluconeogenesis; increasing hepatic β-oxidation; improving insulin sensitivity; and inhibiting adipogenesis and inflammation in adipose tissue [13,14].

These promising, previous results led us to a further investigation of a CEM for fat loss in human subjects. The present trial investigated the efficacy and safety of a CEM on the body fat and blood lipid levels in overweight adults.

## 2. Materials and Methods

### 2.1. Trial Design

This 12-week, randomized, double-blind, placebo-controlled, parallel-group trial was conducted at the Kyung Hee University Hospital and Research Institute of Medical Nutrition of Kyung Hee University in the Republic of Korea. The trial was conducted in accordance with the ethical principles of the Declaration of Helsinki and the Korean Good Clinical Practice guidelines and was approved by the institutional review board of Kyung Hee University Hospital (approval no. KMC IRB 1401-05).

### 2.2. Participants

Adults aged ≥20 and ≤60 years with BMIs from 25 to <30 kg/m^2^ were eligible to participate. Key exclusion criteria were having obesity-related diseases, such as hypertension, diabetes mellitus, and cardiovascular diseases, and taking anti-obesity medication. A full list of the eligibility criteria is provided in Table 1.

### 2.3. Procedures

This trial was designed to provide treatment with either a high or low dose of the CEM or placebo to overweight subjects and evaluate their body fat reduction during 12 weeks of treatment. After a 1-week screening period, eligible participants were randomly assigned in a 1:1:1 ratio to receive either 280 mg or 400 mg of the CEM, or a placebo, administered orally three times a day for 12 weeks. Participants received a 4-week supply of the investigational product at baseline and weeks 4 and 8. At weeks 4, 8, and 12, unused tablets were returned and counted for the evaluation of the participants’ compliances. Following the administration of the CEM or placebo, participants visited the hospital at weeks 4, 8, and 12 (a visit window of ±7 days) and were evaluated for the efficacy and safety of the investigational product. Medical nutrition therapy, which standardized a series of processes, such as nutritional status assessment, diagnosis, nutritional intervention, and evaluation, was conducted by an experienced clinical dietitian. Subjects had their dietary intakes controlled by a registered dietitian and avoided other supplements.

### 2.4. Study Products and Interventions

The CEM is a 30% aqueous ethanol extract of *Crataegus pinnatifida* leaves and *Citrus unshiu* peels. It was produced by a Bulk Good Manufacturing Practice-certified manufacturer according to the method reported by Lee et al. (2016) [14]. The contents of vitexin and narirutin, marker compounds of CEMs, were quantified using the high-performance liquid chromatography analysis reported by Lee et al. (2016) [14]. The batch used for this trial was confirmed to satisfy the established specifications.

The active product was an 800 mg tablet containing either 140 or 200 mg of the CEM, while the placebo tablet contained microcrystalline cellulose instead. The active and placebo tablets were identical in size, shape, and color. Participants were orally administered either 2 active or placebo tablets per dose, three times a day (morning, noon, and evening after meals) for 12 weeks. The daily doses were 840 and 1200 mg for the low- and high-dose CEM treatments, respectively.

### 2.5. Efficacy Outcome Measures

Body fat percentage and fat mass were assessed using dual-energy X-ray absorptiometry (DXA) (Lunar Prodigy, GE Healthcare, Chicago, IL, USA) at baseline and week 12 as the primary outcome measures. Bioelectrical impedance analysis (BIA) (InBody 4.0, Biospace, Seoul, Republic of Korea) was also used to measure the body fat percentage and fat mass at baseline and weeks 4, 8, and 12. Anthropometric parameters, including body weight, BMI, waist and hip circumferences, and waist-to-hip ratio, were measured at baseline and weeks 4, 8, and 12. The waist and hip circumferences were measured at the smallest circumference of the natural waist and at the largest circumference of the buttocks, respectively. Anthropometric parameters were measured in the fasting state with light clothing by the same trained investigator at the same time (±1 h).

Serum levels of lipids, free fatty acids, and adipokines, including adiponectin, leptin, and visfatin, were measured at baseline and week 12. Concentrations of TGs, TC, high-density lipoprotein cholesterol (HDL-C), and low-density lipoprotein cholesterol (LDL-C) were analyzed using an automatic analysis system (Modular Analytics, Roche Diagnostics, Mannheim, Germany) at Green Cross Laboratories (Yongin-Si, Gyeonggi-do, Republic of Korea). Very low-density lipoprotein cholesterol (VLDL-C) concentrations were estimated by using the Friedewald equation, which calculates the VLDL-C by dividing the TGs by 5. Levels of free fatty acids were measured by colorimetry (Cobas 8000, Roche Diagnostics, Mannheim, Germany) using a commercial kit (NEFA-HR (2), Wako Chemicals, Osaka, Japan). Leptin concentrations were measured by a radioimmunoassay (LINCO Research, Inc., St. Charles, MO, USA). Adiponectin and visfatin levels were measured using a commercialized enzyme-linked immunosorbent assay kit (human adiponectin ELISA, R&D Systems, Minneapolis, MN, USA; human visfatin ELISA, Adipogen, Liestal, Switzerland). Blood levels were measured under fasting conditions for at least 12 h.

### 2.6. Safety Outcomes

Safety evaluations were conducted based on adverse events, abnormal laboratory findings, changes in vital signs, and observations from physical examinations. Laboratory analyses, including hematological, serum biochemical, and urine analyses, were conducted at screening, baseline, and week 12. Vital signs were monitored and a physical examination was performed at every scheduled visit.

### 2.7. Sample Size

This study primarily aimed to determine whether the CEM was superior to the placebo in terms of the change from the baseline in the body fat percentage as measured by DXA at 12 weeks. The difference in the mean fat percentage change after the 12 weeks of administration between the CEM and placebo groups was hypothesized to be 1.06% with a standard deviation of 1.40%. To achieve a statistical power of 80% with a significance level of 5%, 29 subjects in each group were required. Given a drop-out rate of 20%, a total of 105 subjects, 35 in each group, were included in this trial.

### 2.8. Statistical Analysis

The outcome data were analyzed by using both intention-to-treat and per-protocol populations. The intention-to-treat population comprised all randomized participants, and the per-protocol population was composed of participants whose primary efficacy assessments were performed according to the protocol without major protocol violations, such as unmet inclusion/exclusion criteria or the use of prohibited medication. Participants whose compliances to treatment were less than 80% were excluded from the per-protocol population. Missing data were replaced using the last-observation-carried-forward method. A one-way analysis of variance analysis with an LSD post hoc test was performed to examine the inter-group differences in the primary and secondary endpoints. Statistical analyses were performed using SPSS 23.0 (SPSS, Inc., Chicago, IL, USA).

## 3. Results

### 3.1. Participants

**Figure 1:**
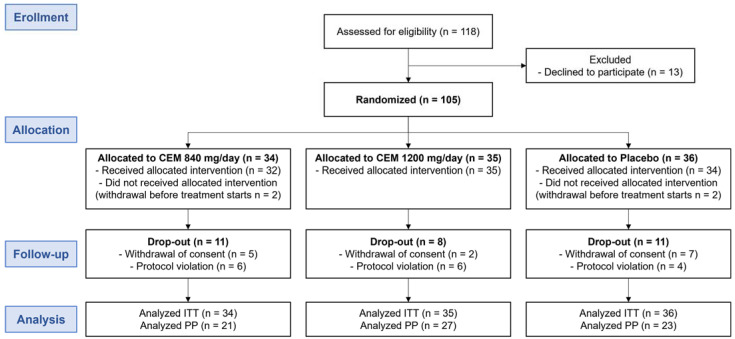
Flow diagram of the study participants.

The trial was conducted from May 2014 to March 2015 and included 105 participants (Figure 1). Overall, 67.6% of the participants completed the trial (61.8%, 77.1%, and 63.9% in the low-dose, high-dose, and placebo groups, respectively), and ≥80% adhered to the CEM treatment or placebo as assigned. There was no treatment discontinuation due to adverse events in any groups.

The socio-demographic and clinical baseline characteristics were generally similar across all groups (Table 2). The mean age of the participants was 31.0 years, most were female (60.0%) and single (60.0%), the mean body weight was 75.4 kg, the mean BMI was 27.1 kg/m^2^, and the mean waist and hip circumferences were 92.1 and 103.5 cm, respectively. At baseline, there were no significant differences between the groups in the DXA and VIA variables, anthropometric parameters, or serum levels of lipids, free fatty acids, and adipokines.

### 3.2. Primary Efficacy Outcomes

**Figure 2:**
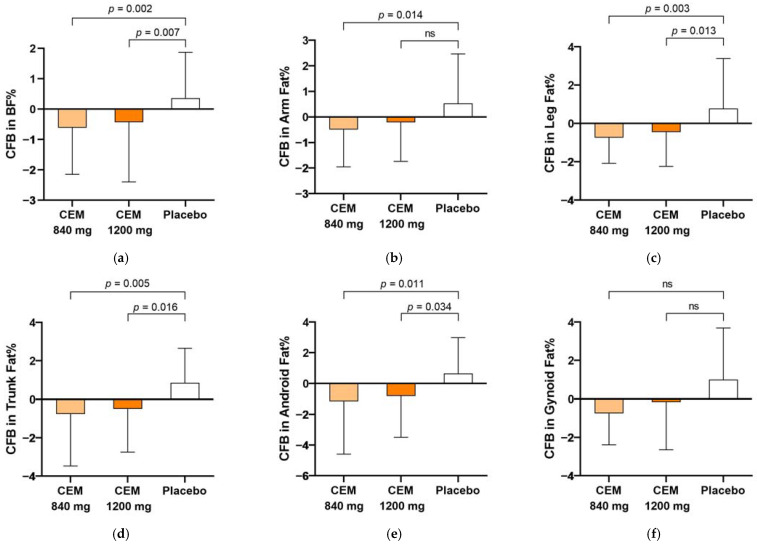

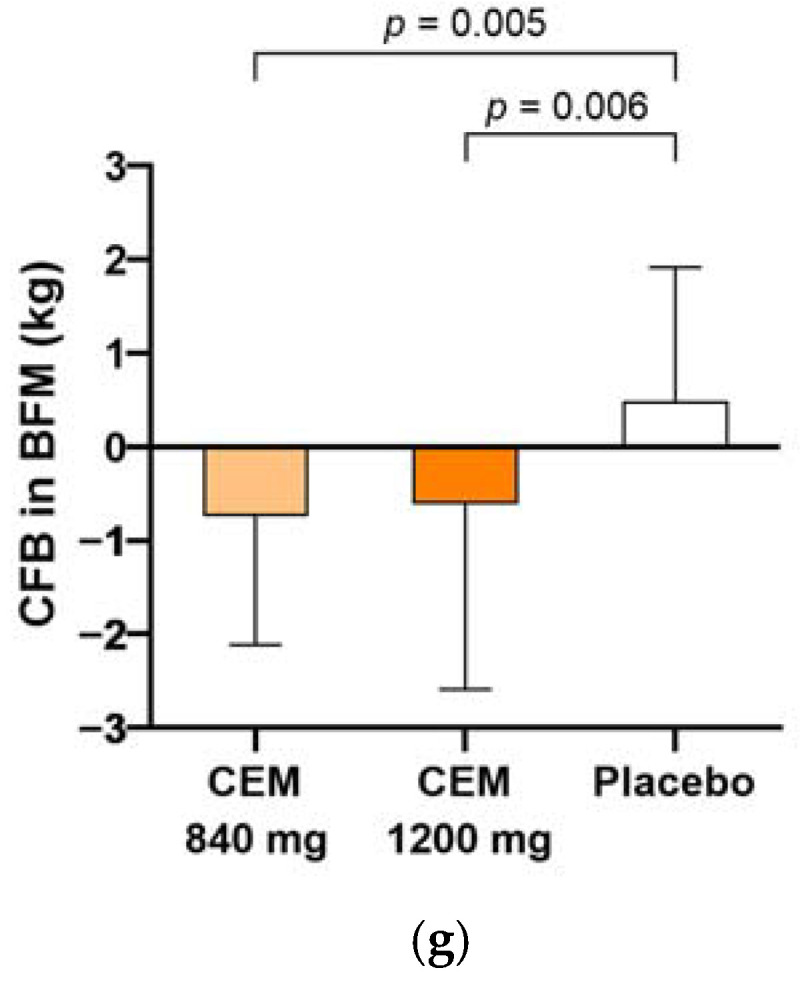
Changes in total and regional body fat percentages (**a**–**f**) and body fat mass (**g**) at week 12, as measured by dual-energy X-ray absorptiometry. The figure illustrates the changes from baseline at week 12 in total body fat percentage (**a**), as well as in the fat percentages of five regions: arms (**b**), legs (**c**), trunk (**d**), android (**e**), and gynoid (**f**). The *p*-value signifies the statistical significance of the differences compared to the placebo, as determined by the LSD post hoc test. Daily dosages of Crataegus Extract Mixture (CEM) were 840 and 1200 mg/day. BF, body fat; BFM, body fat mass; CFB, change from baseline; ns, not significant.

Changes in the fat percentage and fat mass measured by DXA, the primary endpoints of this trial, are shown in Table 3 and Figure 2. The low- and high-dose CEM treatments for 12 weeks significantly reduced the total body fat percentage (*p* = 0.002 and 0.007 vs. placebo, respectively) and fat mass (*p* = 0.005 and 0.006 vs. placebo, respectively). The fat percentages of four regions, the arms, legs, trunk, and android, decreased in the low-dose group (*p* = 0.014, 0.003, 0.005, and 0.011 vs. placebo, respectively), while the leg, trunk, and android fat percentages were reduced in the high-dose group (*p* = 0.013, 0.016, and 0.034 vs. placebo, respectively). The CEM administration did not alter the fat-free mass or lean body mass.

### 3.3. Secondary Efficacy Outcomes

#### 3.3.1. Body Fat Measured by BIA

As shown in Table 4, the BIA revealed significant reductions in the body fat mass after 12 weeks of the low- and high-dose CEM treatments (*p* = 0.002 and 0.005 vs. placebo, respectively).

#### 3.3.2. Anthropometric Parameters

The 12-week changes in the anthropometric parameters are shown in Table 4. The low- and high-dose CEM treatments significantly reduced both the body weight (*p* = 0.021 and 0.011 vs. placebo, respectively) and BMI (*p* = 0.024 and 0.009 vs. placebo, respectively). The waist circumference, hip circumference, and waist-to-hip ratio were not changed by the CEM administration.

#### 3.3.3. Serum Lipid Concentrations

As shown in Table 5, the serum lipid profiles, including the TGs, TC, HDL-C, LDL-C, and VLDL-C, were not changed by the CEM treatment in the intention-to-treat subjects. We performed an additional per-protocol analysis and found that the high dose of the CEM significantly lowered the TG and VLDL-C levels compared to the placebo (Table S1).

#### 3.3.4. Serum Concentrations of Free Fatty Acids and Adipokines

The levels of free fatty acids were not changed by 12 weeks of CEM administration (Table 5). The circulating levels of leptin, the primary adipokine, were significantly decreased in the high-dose CEM group compared to the placebo group (*p* = 0.001). The CEM did not affect the levels of the other two adipokines, adiponectin and visfatin (data not shown).

### 3.4. Safety Outcomes

No adverse events were reported during the CEM intake. There were no significant alterations in the vital signs or hematological and blood biochemical parameters (data not shown).

## 4. Discussion

*C. pinnatifida* and *C. unshiu* are widely consumed for fat loss and weight management in East Asia, but their efficacy has not been studied in well-designed prospective studies. In this double-blind, randomized, controlled trial, overweight adults treated with low and high doses of a CEM achieved significantly greater decreases in their body fat percentages, body fat masses, body weights, and BMIs compared with those treated with the placebo. In addition, the high dose of the CEM significantly lowered the serum leptin levels.

We used three measurement methods, DXA, BIA, and anthropometry, to assess the efficacy of the CEM in reducing body fat. Following both the low- and high-dose treatments with CEM, positive results were observed across all three measurement outcomes. DXA and BIA are commonly employed methods for body composition assessment, with numerous studies reporting a robust correlation between the fat masses obtained by DXA and BIA [15,16]. DXA operates by analyzing the differential attenuations of two distinct X-ray energies, enabling it to distinguish among bone, fat, and lean tissue. It is considered a highly accurate and reproducible method, often referred to as the gold standard in body fat measurement [17]. BIA estimates body fat by measuring the electrical resistance within the body based on the water content, although the results can be affected by various factors [18]. Some research has suggested that BIA might provide inaccurate body fat estimates in obese individuals due to variations in their body water distributions [15]. In general, DXA is believed to offer greater accuracy compared to BIA, with reduced variability. An additional feature of DXA is its ability to quantify regional body fat. In our study, the CEM not only reduced the total body fat percentage but also the fat percentages in specific regions, such as the arms, legs, trunk, and android. Both the DXA and BIA results showed a decrease in the body fat mass, accompanied by reductions in the body weight and BMI. These consistent findings across measurements support the efficacy of CEMs in reducing body fat.

Importantly, the DXA showed that the CEM helped retain lean body mass (LBM) while reducing body fat. LBM accounts for the total body weight minus the body fat mass, including the weight of muscles, bones, body water, etc. In certain diet regimens, the weight loss comes from a reduction in LBM [19]. A decrease in LBM has adverse effects on health, including a decline in resting energy expenditure, increased fatigue, reduced neuromuscular strength, and a higher risk of injury [20]. Moreover, metabolic slowdown due to decreased LBM may predispose individuals to regain weight [21]. Thus, preserving LBM is essential for sustained weight loss and preventing potential health complications. Treatment with the CEM decreased body fat without losing LBM, suggesting CEMs as a potential strategy for weight loss maintenance and body composition improvement.

The leptin levels in the high-dose CEM group decreased by 2.8 ng/mL from a baseline of 16.2 ng/mL, a significant decrease compared to the placebo. Leptin, a hormone primarily secreted by adipose tissue, suppresses hunger and stimulates sympathetic nerve outflow, leading to increased energy expenditure. Leptin is present at high concentrations in overweight and obese individuals who are insensitive to endogenous leptin production, suggesting leptin resistance. Numerous studies have demonstrated that the blood leptin concentration has a strong positive correlation with the body fat mass and BMI in Korean individuals [22,23]. In healthy Korean adults with normal BMIs, the mean serum leptin concentrations have been reported to range from 2 to 5 ng/mL in males and from 5 to 12 ng/mL in females [23,24,25]. In a previous study involving healthy Koreans without medical diseases, the leptin concentrations were approximately 2–3 times higher in those with BMIs of ≥25 kg/m^2^ than those with BMIs of <25 kg/m^2^ [26], which is in line with our results. Our results suggest that CEMs decrease the circulating leptin levels by reducing the body fat mass and can be used to alleviate leptin resistance in overweight individuals.

Adherence to the high-dose CEM treatment up to 12 weeks lowered the TG levels by 20.2% compared to baseline (a decrease of 28.4 mg/dL from 140.9 mg/dL) and lowered the VLDL-C levels, which are calculated as one-fifth of the TG levels. Triglycerides are synthesized primarily in the liver, and adipose tissue from fatty acids originates from dietary or endogenous sources. Triglycerides serve as an important source of energy in the body, but, when excessive, they contribute to an elevated cardiovascular risk. Triglycerides produced by the liver are released into the bloodstream in the form of VLDL particles, which become smaller and transform into intermediate-density lipoprotein and then into LDL, which causes atherosclerosis [27]. Although a TG level of <150 mg/dL is classified as normal, the American Heart Association recommends that a TG level below 100 mg/dL is considered optimal [28]. Our results indicate that the CEM lowered the TG levels closer to the ideal levels in overweight adults, which may ultimately reduce their cardiovascular risk.

CEMs and the herbs that comprise them, *C. pinnatifida* leaf and *C. unshiu* peel, have been reported to exert anti-obesity effects by inhibiting lipid absorption, improving lipid metabolism, and regulating obesity-induced hormonal imbalances and inflammation [9,13,14,29,30,31,32,33]. In preclinical studies using diet-induced-obesity rat models [13,14], CEMs increased the fecal lipid excretion and hepatic β-oxidation and decreased adipogenesis and lipogenesis in both the epididymal adipose tissue and liver. They also inhibited the high-fat-diet-induced increase in the blood insulin levels and the expression of pro-inflammatory cytokines in epididymal fat [14]. These mechanisms might contribute to reductions in body fat and decreases in the blood leptin, TG, and VLDL-C levels after CEM treatment. In addition, supporting our results that the leptin and TG levels significantly decreased in the high-dose CEM group, the leptin expression in adipose tissue and the serum TG levels were decreased following CEM treatment in obese rats [13].

It is believed that flavonoids, which are abundantly contained in *C. pinnatifida* leaves and *C. unshiu* peels, mainly contribute to the anti-obesity effects of CEMs. The flavonoids in *C. pinnatifida* leaves, predominantly *C*-glycosyl flavones derived from apigenin and luteolin, such as vitexin, vitexin rhamnoside, and vitexin glucoside, along with flavanol glycoside derivatives of quercetin and kaempferol, such as hyperoside, isoquercetrin, and rutin, are widely known to be responsible for the lipid-regulating and anti-atherosclerosis effects of these leaves [34,35]. The flavonoid fraction of *C. pinnatifida* leaf has been reported to decrease the triglyceride absorption in olive oil-loaded mice partially via pancreatic lipase inhibition [9,36]. The flavonoids in *C. pinnatifida* leaves have shown hypolipidemic activity through multiple mechanisms, including inhibiting cholesterol absorption, adipogenesis, and hepatic lipogenesis and increasing hepatic fatty acid oxidation [37]. Citrus flavonoids, especially naringin, naringenin, nobiletin, and hesperidin, are well known to reverse obesity and related metabolic abnormalities through the regulation of lipid metabolism, improvement in insulin resistance, and suppression of inflammatory responses in adipose tissue [38,39,40].

The CEM at both low and high doses reduced the body fat percentage, body fat mass, body weight, and BMI, demonstrating that the effective dose range for body fat loss and weight management is from 840 to 1200 mg/day. Considering the extraction yield, 840–1200 mg/day of CEM corresponds to 3–6 g of *C. pinnatifida* leaves/day and 2–4 g of *C. unshiu* peels/day. These raw-material weight ranges correspond to the traditional dosages of both herbs [41]. A CEM showed anti-obesity effects when administered at a dose of 100 mg/kg/day in rats fed a high-fat diet [13,14]. When converted to a human equivalent dose, this rat dose is equivalent to 16 mg per kg per day, which equates to 1200 mg/day for a person weighing 75 kg. We established 1200 mg/day as the high dose and, considering the traditional dosage, we set 70% of the high dose (840 mg/day) as the low dose. Significant reductions in the serum leptin, TG, and VLDL-C levels were observed in the high-dose group but not in the low-dose group, indicating the greater efficacy of the high dose in regulating these levels. Furthermore, the decreases in the TG and VLDL-C levels among the per-protocol subjects who completed 12 weeks of high-dose treatment indicate that high compliance with CEM treatment is needed to improve blood lipid profiles.

No adverse events were observed during the CEM treatment. The leaves of the Crataegus genus (commonly called hawthorn) and *C. unshiu* peels have been established as safe [12,42]. In the previous clinical study, no adverse events were reported following the administration of *C. pinnatifida* leaf extracts [43]. Among overweight or obese patients, treatment with *C. unshiu* peel was well tolerated, with only minor adverse effects that resolved spontaneously [12]. Additionally, water extracts of *C. unshiu* peel showed no genotoxicity and exhibited low acute and subchronic toxicity in rats [44]. Our safety outcome, together with previously reported safety profiles, indicates that CEMs are safe for use in overweight adults aiming for body fat and weight reduction.

As our study recruited Asian participants with BMIs of 25–30 and no medical problems, and the average age of the participants was 31.0 years, it is challenging to extrapolate our study results to older age groups, other ethnicities, or people with various medical difficulties. CEMs have been developed as functional food ingredients intended to reduce the body fat in individuals who are overweight but do not have significant medical illnesses, often referred to as sub-healthy or mildly unhealthy individuals. To explore their potential for use as medicine, research that includes older or other ethnic populations, or those with medical difficulties, could be considered for future study directions.

## 5. Conclusions

This double-blind, randomized, controlled study suggests that CEMs are effective and safe for reducing the body fat and weight and improving the lipid and leptin profiles in overweight subjects. CEMs are of value to prevent the progression to mild-to-moderate obesity, hypertriglyceridemia, and leptin resistance in overweight individuals.

## Figures and Tables

**Table 1:** Eligibility criteria.

Inclusion and Exclusion Criteria
**Inclusion criteria**
Participants who met all of the following criteria were eligible for inclusion:
1. Men and women aged ≥20 and ≤60 years;
2. A body mass index from 25 to <30 kg/m^2^;
3. Participants who voluntarily decided to participate and signed the informed consent.
**Exclusion criteria**
Participants were excluded if any of the following criteria applied:
1. A systolic blood pressure of ≥160 mmHg or diastolic blood pressure of ≥100 mmHg; hypertensive patients taking diuretics;
2. A fasting blood glucose level of ≥126 mg/dL or random blood glucose level of ≥200 mg/dL; participants with diabetes mellitus taking oral hypoglycemic drugs or insulin;
3. Diseases of the heart, kidney, liver, or thyroid, or cerebrovascular diseases;
4. Gallbladder diseases, gastrointestinal diseases, gout, or porphyria;
5. Mental Disorders such as depression, schizophrenia, alcoholism, drug addiction, etc.;
6. Taking anti-obesity drugs;
7. Pregnant or breastfeeding;
8. Attended weight loss program or consumed diet food within the last 30 days;
9. Persons unable to exercise due to severe musculoskeletal disorders;
10. Diagnosed with and treated for cancer within the last 5 years;
11. Asthma and other allergic diseases;
12. A history of surgery within the last 6 months;
13. A history of drug or alcohol addiction;
14. Persons who have participated in another clinical trial within the last 3 months;
15. Illiterate persons or persons with a limited ability to read;
16. Persons considered to be inadequate for participation due to other reasons.

**Table 2:** Socio-demographic and clinical characteristics of participants at baseline.

Characteristic	CEM 840 mg/day (*n* = 34)	CEM 1200 mg/day (*n* = 35)	Placebo (*n* = 36)	Total (*n* = 105)
Age (years)	31.0 (10.4) ^1^	31.3 (9.8)	30.8 (9.2)	31.0 (9.7)
Sex				
Male	13 (38.2)	16 (45.7)	13 (36.1)	42 (40.0)
Female	21 (61.8)	19 (54.3)	23 (63.9)	63 (60.0)
Marital status				
Married	12 (35.3)	15 (42.9)	17 (47.2)	42 (40.0)
Single	22 (64.7)	20 (57.1)	19 (52.8)	63 (60.0)
Regular exercise				
Yes	15 (44.1)	14 (40.0)	17 (47.2)	46 (43.8)
No	19 (55.9)	21 (60.0)	19 (52.8)	59 (56.2)
Smoking status				
Never	26 (76.5)	26 (74.3)	28 (77.8)	80 (76.2)
Ex-smoker	3 (8.8)	2 (5.7)	1 (2.8)	6 (5.7)
Current smoker	5 (14.7)	7 (20.0)	7 (19.4)	19 (18.1)
Drinking status				
Never	11 (32.4)	15 (42.9)	14 (38.9)	40 (38.0)
Drinker	23 (67.6)	20 (57.1)	22 (61.1)	65 (62.0)
**DXA variables**				
Fat percentage (%)				
Total body fat	38.1 (7.3)	37.6 (5.5)	37.1 (7.5)	37.6 (7.6)
Arms	36.1 (10.5)	35.2 (9.1)	34.6 (10.5)	35.3 (10.0)
Legs	35.9 (10.1)	34.8 (7.4)	34.0 (8.5)	34.9 (8.3)
Trunk	41.9 (6.6)	41.8 (5.0)	41.5 (7.5)	41.7 (6.3)
Android	46.7 (7.2)	47.8 (4.8)	47.4 (7.5)	47.3 (9.6)
Gynoid	43.7 (10.4)	43.0 (7.6)	42.5 (9.3)	41.7 (6.5)
Body fat mass (kg)	26.3 (4.8)	26.9 (3.1)	26.5 (5.7)	26.6 (4.6)
Fat-free mass (kg)	45.1 (9.1)	48.3 (9.2)	48.0 (9.3)	47.2 (9.2)
Lean body mass (kg)	43.4 (9.0)	45.3 (8.6)	45.1 (8.8)	47.8 (32.0)
**BIA variables**				
Body fat mass (kg)	25.2 (4.5)	25.1 (3.2)	24.9 (5.3)	25.1 (4.4)
Body fat percentage (%)	34.4 (6.5)	33.5 (5.9)	33.0 (7.2)	33.7 (6.5)
**Anthropometric parameters**				
Body weight (kg)	73.9 (9.7)	76.1 (9.3)	76.3 (9.5)	75.4 (9.4)
Body mass index (kg/m^2^)	27.1 (1.6)	27.1 (1.5)	27.2 (1.5)	27.1 (1.5)
Waist circumference (cm)	92.3 (7.0)	92.5 (6.4)	91.4 (6.3)	92.1 (6.5)
Hip circumference (cm)	103.1 (4.5)	103.8 (2.9)	103.6 (5.1)	103.5 (4.2)
Waist-to-hip ratio	0.9 (0.1)	0.9 (0.1)	0.9 (0.1)	0.9 (0.1)
**Serum levels**				
Triglycerides (mg/dL)	133.1 (109.1)	130.1 (76.5)	102.9 (46.4)	121.7 (80.9)
Total cholesterol (mg/dL)	187.3 (35.8)	188.5 (31.1)	184.8 (33.3)	186.9 (32.9)
HDL-C (mg/dL)	55.8 (14.4)	57.3 (11.6)	56.7 (13.6)	56.6 (13.1)
LDL-C (mg/dL)	113.3 (29.7)	115.3 (27.9)	114.2 (29.1)	114.3 (28.6)
VLDL-C (mg/dL)	26.6 (21.8)	26.0 (15.3)	20.6 (9.3)	24.3 (16.2)
Free fatty acids (µEq/L)	668.3 (238.4)	568.5 (135.1)	586.8 (216.4)	606.3 (202.8)
Leptin (ng/mL)	16.5 (10.6)	16.2 (10.3)	14.6 (10.3)	15.7 (10.3)

^1^ Data are presented as mean (SD) or no. (%). BIA, bioelectrical impedance analysis; DXA, dual-energy X-ray absorptiometry; HDL-C, high-density lipoprotein cholesterol; LDL-C, low-density lipoprotein cholesterol; VLDL-C, very low-density lipoprotein cholesterol.

**Table 3:** Body composition measured by dual-energy X-ray absorptiometry (DXA) after 12 weeks of CEM administration.

DXA Variables ^1^	CEM 840 mg/day (*n* = 34)	CEM 1200 mg/day (*n* = 35)	Placebo (*n* = 36)	*p* Value ^2^
Changes in fat percentages (%)				
Total body fat	−0.7 (1.8) ^3,^*	−0.4 (1.8) *	0.8 (1.8)	**0.003**
Arms	−0.5 (1.5) *	−0.2 (1.5)	0.5 (1.9)	**0.039**
Legs	−0.7 (1.3) *	−0.5 (1.8) *	0.8 (2.6)	**0.006**
Trunk	−0.8 (2.7) *	−0.5 (2.3) *	0.9 (1.8)	**0.010**
Android	−1.1 (3.4) *	−0.8 (2.7)*	0.7 (2.3)	**0.025**
Gynoid	−0.7 (1.6)	−0.1 (2.5)	1.0 (2.7)	0.116
Changes in measures				
Body fat mass (kg)	−0.6 (1.6) *	−0.6 (1.8) *	0.8 (1.4)	**0.000**
Fat-free mass (kg)	1.1 (5.3)	−0.2 (1.2)	−0.3 (2.0)	0.177
Lean body mass (kg)	0.1 (0.9)	−0.2 (1.2)	0.0 (2.4)	0.770

^1^ All changes are from baseline to week 12. ^2^ One-way analysis of variance. ^3^ All values are means (SDs). * Statistically significant difference vs. placebo in LSD post hoc test. Bold values indicate statistical significance.

**Table 4:** Body fat measured by bioelectrical impedance analysis (BIA) and anthropometry after 12 weeks of CEM administration.

Endpoints ^1^	CEM 840 mg/day (*n* = 34)	CEM 1200 mg/day (*n* = 35)	Placebo (*n* = 36)	*p* Value ^2^
Changes in BIA variables				
Body fat mass (kg)	−0.7 (1.4) ^3,^*	−0.6 (2.0) *	0.5 (1.4)	**0.003**
Body fat percentage (%)	−0.6 (1.5)	−0.4 (2.0)	0.4 (1.5)	0.556
Changes in anthropometric parameters				
Body weight (kg)	−0.8 (1.8) *	−0.9 (2.2) *	0.4 (1.9)	**0.020**
Body mass index (kg/m^2^)	−0.3 (0.7) *	−0.3 (0.9) *	0.1 (0.7)	**0.019**
Waist circumference (cm)	−1.3 (3.3)	−1.3 (3.8)	0.5 (3.5)	0.066
Hip circumference (cm)	−0.3 (1.7)	−1.0 (2.7)	−0.1 (2.6)	0.343
Waist-to-hip ratio	0.0 (0.0)	0.0 (0.0)	0.0 (0.0)	0.111

^1^ All changes are from baseline to week 12. ^2^ One-way analysis of variance. ^3^ All values are means (SDs). * Statistically significant difference vs. placebo in LSD post hoc test. Bold values indicate statistical significance.

**Table 5:** Serum levels of lipids, free fatty acids, and leptin after 12 weeks of CEM treatment.

Endpoints ^1^	CEM 840 mg/day (*n* = 34)	CEM 1200 mg/day (*n* = 35)	Placebo (*n* = 36)	*p* Value ^2^
Changes in lipid profiles (mg/dL)				
Triglycerides	−18.6 (43.9) ^3^	−19.4 (49.9)	0.6 (32.5)	0.092
Total cholesterol	−3.4 (16.6)	−1.1 (21.8)	1.8 (25.6)	0.623
HDL-C	−0.1 (5.8)	1.4 (7.9)	0.2 (8.0)	0.674
LDL-C	0.1 (16.4)	2.5 (19.7)	4.1 (22.0)	0.710
VLDL-C	−3.7 (8.8)	−3.9 (10.0)	0.1 (6.5)	0.091
Changes in measures				
Free fatty acids (µEq/L)	−33.3 (234.3)	−27.5 (161.5)	−23.1 (193.0)	0.978
Leptin (ng/mL)	0.1 (4.9)	−2.8 (7.4) *	2.2 (6.0)	**0.005**

^1^ All changes are from baseline to week 12. ^2^ One-way analysis of variance. ^3^ All values are means (SDs). * Statistically significant difference vs. placebo in LSD post hoc test. Bold values indicate statistical significance. HDL-C, high-density lipoprotein cholesterol; LDL-C, low-density lipoprotein cholesterol; VLDL-C, very low-density lipoprotein cholesterol.

## Data Availability

Data sharing is not available for this study due to ethics.

## Supplementary Materials

The following supporting information can be downloaded at https://www.mdpi.com/article/10.3390/nu16040494/s1, Table S1: Serum lipid concentrations of per-protocol population after 12 weeks of CEM treatment.

## References

[1] Boutari C., Mantzoros C.S. (2022). A 2022 update on the epidemiology of obesity and a call to action: As its twin COVID-19 pandemic appears to be receding, the obesity and dysmetabolism pandemic continues to rage on. Metabolism.

[2] Korea Disease Control and Prevention Agency (2022). The Korea National Health and Nutrition Examination Survey 2021.

[3] Jin X., Qiu T., Li L., Yu R., Chen X., Li C., Proud C.G., Jiang T. (2023). Pathophysiology of obesity and its associated diseases. Acta Pharm. Sin. B.

[4] Hou J., He C., He W., Yang M., Luo X., Li C. (2020). Obesity and Bone Health: A Complex Link. Front. Cell Dev. Biol..

[5] Chakhtoura M., Haber R., Ghezzawi M., Rhayem C., Tcheroyan R., Mantzoros C.S. (2023). Pharmacotherapy of obesity: An update on the available medications and drugs under investigation. eClinicalMedicine.

[6] Hendricks E.J. (2017). Off-label drugs for weight management. Diabetes Metab. Syndr. Obes..

[7] Onakpoya I.J., Heneghan C.J., Aronson J.K. (2016). Post-marketing withdrawal of anti-obesity medicinal products because of adverse drug reactions: A systematic review. BMC Med..

[8] Guo J., Tao H., Cao Y., Ho C.T., Jin S., Huang Q. (2016). Prevention of Obesity and Type 2 Diabetes with Aged Citrus Peel (Chenpi) Extract. J. Agric. Food Chem..

[9] Wang T., An Y., Zhao C., Han L., Boakye-Yiadom M., Wang W., Zhang Y. (2011). Regulation effects of Crataegus pinnatifida leaf on glucose and lipids metabolism. J. Agric. Food Chem..

[10] Hu M., Zeng W., Tomlinson B. (2014). Evaluation of a crataegus-based multiherb formula for dyslipidemia: A randomized, double-blind, placebo-controlled clinical trial. Evid. Based Complement. Alternat Med..

[11] Assini J.M., Mulvihill E.E., Huff M.W. (2013). Citrus flavonoids and lipid metabolism. Curr. Opin. Lipidol..

[12] Kang S., Song S., Lee J., Chang H., Lee S. (2018). Clinical Investigations of the Effect of Citrus unshiu Peel Pellet on Obesity and Lipid Profile. Evid. Based Complement. Altern. Med..

[13] Lee Y.H., Kim Y.S., Song M., Lee M., Park J., Kim H. (2015). A Herbal Formula HT048, Citrus unshiu and Crataegus pinnatifida, Prevents Obesity by Inhibiting Adipogenesis and Lipogenesis in 3T3-L1 Preadipocytes and HFD-Induced Obese Rats. Molecules.

[14] Lee Y.H., Jin B., Lee S.H., Song M., Bae H., Min B.J., Park J., Lee D., Kim H. (2016). Herbal Formula HT048 Attenuates Diet-Induced Obesity by Improving Hepatic Lipid Metabolism and Insulin Resistance in Obese Rats. Molecules.

[15] Achamrah N., Colange G., Delay J., Rimbert A., Folope V., Petit A., Grigioni S., Dechelotte P., Coeffier M. (2018). Comparison of body composition assessment by DXA and BIA according to the body mass index: A retrospective study on 3655 measures. PLoS ONE.

[16] Cruz Rivera P.N., Goldstein R.L., Polak M., Lazzari A.A., Moy M.L., Wan E.S. (2022). Performance of bioelectrical impedance analysis compared to dual X-ray absorptiometry (DXA) in Veterans with COPD. Sci. Rep..

[17] Bazzocchi A., Ponti F., Albisinni U., Battista G., Guglielmi G. (2016). DXA: Technical aspects and application. Eur. J. Radiol..

[18] Dehghan M., Merchant A.T. (2008). Is bioelectrical impedance accurate for use in large epidemiological studies?. Nutr. J..

[19] Ardavani A., Aziz H., Smith K., Atherton P.J., Phillips B.E., Idris I. (2021). The Effects of Very Low Energy Diets and Low Energy Diets with Exercise Training on Skeletal Muscle Mass: A Narrative Review. Adv. Ther..

[20] Willoughby D., Hewlings S., Kalman D. (2018). Body Composition Changes in Weight Loss: Strategies and Supplementation for Maintaining Lean Body Mass, a Brief Review. Nutrients.

[21] Johannsen D.L., Knuth N.D., Huizenga R., Rood J.C., Ravussin E., Hall K.D. (2012). Metabolic slowing with massive weight loss despite preservation of fat-free mass. J. Clin. Endocrinol. Metab..

[22] Kim M., Hong M., Kang Y., Choi Y., Kim S., Kim J., Cho K., Kim W. (2003). The Correlation between Plasma Leptin Concentration and Adiposity in Obesity. Korean J. Fam. Med..

[23] Shin J., Nam S., Na S., Kim E., Kim K., Cha B., Song Y., Lim S., Lee H., Huh K. (1998). Serum immunoreactive-leptin concentrations and its relation to adiposity and other biochemical parameters in Korean Males. Endocrinol. Metab..

[24] Shim D., Kim S., Kim S., Choi Y., Jeong U., Lee H., Choi H., Kim J., Lee S. (1999). Serum leptin concentration in diabetic and nondiabetic Koreans. J. Obes. Metab. Syndr..

[25] Kim D., Kim N., Shin D., Kim S., Choi K., Kim J., Shin C., Lee S., Baik S., Choi D. (2002). Plasma Leptin Concentration, Obesity, and Insulin Resistance in Healthy Korean Population. Diabetes Metab. J..

[26] Kim J., Shin H., Jeong I., Cho S., Min S., Lee S., Park C., Oh K., Hong E., Kim H. (2005). The relationship of adiponectin, leptin and ghrelin to insulin resistance and cardiovascular risk factors in human obesity. Korean J. Med..

[27] Heeren J., Scheja L. (2021). Metabolic-associated fatty liver disease and lipoprotein metabolism. Mol. Metab..

[28] Miller M., Stone N.J., Ballantyne C., Bittner V., Criqui M.H., Ginsberg H.N., Goldberg A.C., Howard W.J., Jacobson M.S., Kris-Etherton P.M. (2011). Triglycerides and cardiovascular disease: A scientific statement from the American Heart Association. Circulation.

[29] Pang X., Wang M., Wang S.-Y., Zhang J., Du Y.-P., Zhao Y., Zheng X.-H., Ma B.-P. (2021). Phenolic compounds from the leaves of Crataegus pinnatifida Bge. var. major N.E.Br. And their lipid-lowering effects. Bioorg. Med. Chem. Lett..

[30] Hu H., Weng J., Cui C., Tang F., Yu M., Zhou Y., Shao F., Zhu Y. (2022). The Hypolipidemic Effect of Hawthorn Leaf Flavonoids through Modulating Lipid Metabolism and Gut Microbiota in Hyperlipidemic Rats. Evid. Based Complement. Altern. Med..

[31] Zeng S.L., Li S.Z., Lai C.J., Wei M.Y., Chen B.Z., Li P., Zheng G.D., Liu E.H. (2018). Evaluation of anti-lipase activity and bioactive flavonoids in the Citri Reticulatae Pericarpium from different harvest time. Phytomedicine.

[32] Jung H.K., Jeong Y.S., Park C.-D., Park C.-H., Hong J.-H. (2011). Inhibitory effect of citrus peel extract on lipid accumulation of 3T3-L1 adipocytes. J. Korean Soc. Appl. Biol. Chem..

[33] Lee S.-Y., Kim M.-H., Bae C.-S., Choi H.J., Ma E.H., Park S.-J., Cho S.-S., Park D.-H. (2023). Unripe Citrus unshiu peel inhibited pre-adipocyte’s differentiation via leptin-PPARγ/FAS pathway and pro-inflammatory cytokines’ release. J. Funct. Foods.

[34] Wu J., Peng W., Qin R., Zhou H. (2014). Crataegus pinnatifida: Chemical constituents, pharmacology, and potential applications. Molecules.

[35] Shu X., Zhang W., Liu Y., Ye X., Chen K., Li X., Cao Y. (2023). The chemistry, stability and health effects of phenolic compounds in cultivated hawthorn (*Crataegus pinnatifida* var. major): A review. Food Qual. Saf..

[36] Tao W., Deqin Z., Yuhong L., Hong L., Zhanbiao L., Chunfeng Z., Limin H., Xiumei G. (2010). Regulation effects on abnormal glucose and lipid metabolism of TZQ-F, a new kind of Traditional Chinese Medicine. J. Ethnopharmacol..

[37] Wu M., Liu L., Xing Y., Yang S., Li H., Cao Y. (2020). Roles and Mechanisms of Hawthorn and Its Extracts on Atherosclerosis: A Review. Front. Pharmacol..

[38] Nakajima V.M., Macedo G.A., Macedo J.A. (2014). Citrus bioactive phenolics: Role in the obesity treatment. LWT-Food Sci. Technol..

[39] Lu K., Yip Y.M. (2023). Therapeutic Potential of Bioactive Flavonoids from Citrus Fruit Peels toward Obesity and Diabetes Mellitus. Future Pharmacol..

[40] Alam M.A., Subhan N., Rahman M.M., Uddin S.J., Reza H.M., Sarker S.D. (2014). Effect of citrus flavonoids, naringin and naringenin, on metabolic syndrome and their mechanisms of action. Adv. Nutr..

[41] Editorial Board of Zhong Hua Ben Cao (1999). Zhong Hua Ben Cao.

[42] Tassell M.C., Kingston R., Gilroy D., Lehane M., Furey A. (2010). Hawthorn (*Crataegus* spp.) in the treatment of cardiovascular disease. Pharmacogn. Rev..

[43] Xu Z., Guo X., Yuan X., Ren X., Pan C. (1991). Study on utilizing the natural resources of Crataegus pinnatifida leaf. Heilongjiang J. Tradit. Chin. Med..

[44] Park H., Hwang Y.H., Choi J.G., Ma J.Y. (2018). In vitro and in vivo evaluation of systemic and genetic toxicity of Citrus unshiu peel. J. Ethnopharmacol..

