# Supplementary material for: Efficacy of Crataegus Extract Mixture on Body Fat and Lipid Profiles in Overweight Adults: A 12-Week, Randomized, Double-Blind, Placebo-Controlled Trial
Source: Nutrients. 2024 Feb 8;16(4):494. doi: 10.3390/nu16040494 (PMC10892674; doi:10.3390/nu16040494)
Supplement: Supplementary file 1 [file nutrients-16-00494-s001.zip › nutrients-2719804-supplementary.pdf]

Table S1. Serum lipids concentrations of per-protocol population after 12 weeks of CEM treatment.

**Change in Lipid Profiles** [1]	**CEM 840 mg/day (N = 21)**	**CEM 1,200 mg/day (N = 27)**	**Placebo (N = 23)**	***P* value** [2]
Triglycerides (mg/dL)	−24.1 (11.0) [3]	**−28.4 (9.7) ***	4.3 (10.2)	**0.049**
Total cholesterol (mg/dL)	−5.9 (5.8)	−0.8 (5.1)	4.9 (5.3)	0.387
HDL-C (mg/dL)	−1.0 (2.0)	2.4 (1.7)	0.6 (1.8)	0.437
LDL-C (mg/dL)	−0.6 (5.2)	3.8 (4.6)	7.8 (4.8)	0.497
VLDL-C mg/dL)	−4.8 (2.2)	**−5.7 (1.9) ***	0.9 (2.0)	**0.049**

[1] All changes are from baseline to week 12.

[2] Analysis of covariance adjusted for intake.

[3] All values are mean (SD).

* Statistically significantly difference vs. placebo by LSD *post hoc* test.

Bold values indicate statistical significance.

HDL-C, high-density lipoprotein cholesterol; LDL-C, low-density lipoprotein cholesterol; VLDL-C, very low-density lipoprotein cholesterol.
